# Recruitment of Glycosyl Hydrolase Proteins in a Cone Snail Venomous Arsenal: Further Insights into Biomolecular Features of *Conus* Venoms

**DOI:** 10.3390/md10020258

**Published:** 2012-01-31

**Authors:** Aude Violette, Adrijana Leonardi, David Piquemal, Yves Terrat, Daniel Biass, Sébastien Dutertre, Florian Noguier, Frédéric Ducancel, Reto Stöcklin, Igor Križaj, Philippe Favreau

**Affiliations:** 1 Atheris Laboratories, Case postale 314, CH-1233 Bernex-Geneva, Switzerland; Email: daniel.biass@atheris.ch (D.B.); reto.stocklin@atheris.ch (R.S.); philippe.favreau@atheris.ch (P.F.); 2 Department of Molecular and Biomedical Sciences, Jožef Stefan Institute, Jamova 39, 1000 Ljubljana, Slovenia; Email: adrijana.leonardi@ijs.si (A.L.); igor.krizaj@ijs.si (I.K.); 3 Skuldtech, Cap Delta, 1682, rue de la Valsière, 34790 Grabels, France; Email: piquemal@skuldtech.com (D.P.); noguier@skuldtech.com (F.N.); 4 CEA-Saclay, 91919 Gif sur Yvette Cedex, France; Email: yves.terrat@yahoo.fr (Y.T.); frederic.ducancel@cea.fr (F.D.)

**Keywords:** *Conus* venom, hyaluronidase, proteomics, transcriptomics, glycosylation

## Abstract

Cone snail venoms are considered an untapped reservoir of extremely diverse peptides, named conopeptides, displaying a wide array of pharmacological activities. We report here for the first time, the presence of high molecular weight compounds that participate in the envenomation cocktail used by these marine snails. Using a combination of proteomic and transcriptomic approaches, we identified glycosyl hydrolase proteins, of the hyaluronidase type (Hyal), from the dissected and injectable venoms (“injectable venom” stands for the venom variety obtained by milking of the snails. This is in contrast to the “dissected venom”, which was obtained from dissected snails by extraction of the venom glands) of a fish-hunting cone snail, *Conus consors* (*Pionoconus* clade). The major Hyal isoform, Conohyal-Cn1, is expressed as a mixture of numerous glycosylated proteins in the 50 kDa molecular mass range, as observed in 2D gel and mass spectrometry analyses. Further proteomic analysis and venom duct mRNA sequencing allowed full sequence determination. Additionally, unambiguous segment location of at least three glycosylation sites could be determined, with glycans corresponding to multiple hexose (Hex) and *N*-acetylhexosamine (HexNAc) moieties. With respect to other known Hyals, Conohyal-Cn1 clearly belongs to the hydrolase-type of Hyals, with strictly conserved consensus catalytic donor and positioning residues. Potent biological activity of the native Conohyals could be confirmed in degrading hyaluronic acid. A similar Hyal sequence was also found in the venom duct transcriptome of *C. adamsonii* (*Textilia* clade), implying a possible widespread recruitment of this enzyme family in fish-hunting cone snail venoms. These results provide the first detailed Hyal sequence characterized from a cone snail venom, and to a larger extent in the Mollusca phylum, thus extending our knowledge on this protein family and its evolutionary selection in marine snail venoms.

## 1. Introduction

A remarkable feature of cone snail venoms is their high content of small, usually disulfide-rich conopeptides, exhibiting various neuropharmacological properties [1]. To date, about 30 distinct molecular receptor types or sub-types have been found to be modulated by more than 15 conopeptide families defined by structural patterns [2]. Some conopeptides are being investigated as potential drugs in pre-clinical and clinical trials and one of them, Prialt, was even brought to the market for the treatment of severe chronic pain [3,4]. In the context of this wealth of information on conopeptides, knowledge of the large proteins included in the cone snail venom cocktail is rather limited [5]. In contrast, a multitude of proteins have been described over the last decades in other venomous animals, such as snakes, spiders, scorpions, hymenopters and sea anemones [6].

A few studies have reported the presence of hydrolases [7], including acetylcholinesterase [8] and phosphodiesterase [9], in cone snail venoms, but only one cysteine-rich secretory protein (CRISP-like) was isolated with full sequence characterization [10], although this initial finding was recently questioned [11]. Several other families of enzymes characterized from cone snail venom ducts have been reported, including a vitamin K-dependent carboxylase [12], peptidylprolyl cis-trans isomerases [13], and several protein disulfide isomerases [14,15,16,17]. While implying considerable impact in venom production, post-translational modification and trafficking, none of these enzymes is considered part of the venomous arsenal intended for prey capture or digestion. These proteins have not been isolated from injectable venom itself (which can be obtained by milking of live specimen), but only from the whole venom gland, either by molecular biology or at the protein level from dissected venom ducts. So far, only one protein has been identified in the injectable venom of *C. purpurascens*. It is an 83-residue cysteine-rich sequence for which the target is currently unknown [18].

The CRISP-like protein and conodipine-M, a phospholipase A_2_ isolated from *C. magus* dissected venom duct, are the only enzyme families from cone snail venom characterized to date that have a possible venomous function [19]. In addition to their enzymatic activity allowing tissue disruption in the prey for digestion, some families of snake venom phospholipase A_2_ have evolved to exhibit neurotoxic functions that directly participate in the immobilization of the prey during capture [20]. However, there is currently no evidence of a phospholipase A_2_ from milked *Conus* venom being injected into the prey, thus questioning its role in prey capture.

In order to identify high molecular mass proteins that could participate in the envenomation process of a cone snail, a series of analytical techniques were applied to the venom within the venom duct as well as to the venom injected by a fish-hunting cone snail species. These experiments led to the identification and sequence characterization of the first hyaluronidases (Hyals) in a cone snail venom. Hyals can be divided into four main groups, including the hydrolase-type (mainly from vertebrates), the lyase-type (from bacteria) and two other poorly known groups, mainly described in leeches/crustaceans and fungi, respectively [21]. These enzymes cleave a glycosidic bond present in hyaluronan and/or chondroitin, which are part of the extracellular matrix of most organisms.

Using a combination of proteomic and transcriptomic analyses, one Hyal component, termed Conohyal-Cn1, was found to be expressed in the whole venom duct, and also detected in the venom duct lumen and in the injectable venom of *C. consors*. By sharing common features with other hydrolase-type Hyals, including sequence similarity, key residue conservation and glycosylation sites, Conohyal-Cn1 clearly belongs to the hydrolase-type class of enzymes, and provides the first sequence description of this protein family from the Mollusca phylum. In addition, a similar Hyal transcript was discovered from the venom duct transcriptome of another species, *C. adamsonii*, thus underlying the possible widespread occurrence of this enzyme family in cone snail venoms.

## 2. Results and Discussion

### 2.1. Discovery of Hyaluronidases in Cone Snail Venom

**Figure 1:**
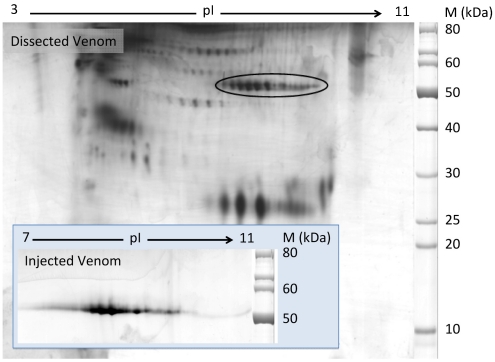
Two-dimensional gel electrophoresis of *C. consors* dissected and injectable venoms. IEF was performed under denaturing conditions using a 3–11 NL IPG strip followed by 10% SDS-PAGE in Tris/Taurine buffer in a second dimension. After silver staining and identification by ESI-MS/MS, 10 and 8 Hyal isoforms were identified in DV and IV respectively. All the DV Hyals are located in the encircled region. The IV Hyal spots are shown in the inset at the bottom left.

The two-dimensional gel electrophoresis (2-DE) of *C. consors* dissected and injectable venoms (DV and IV, respectively) were performed on analytical (10 μg) and semi-preparative levels (350 μg, DV only). A typical DV 2-DE pattern is shown in Figure 1, where 95 well-defined protein spots display relative molecular masses ranging from 15 to 90 kDa under reducing conditions. The 2-DE pattern of IV was much less complex, yet revealing nine distinct spots. Protein spots with relative molecular masses in the range of 50 kDa and basic isoelectric points (pI, encircled in Figure 1) were identified as Hyals by reduction and alkylation followed by in-gel proteolytic digestion, tandem mass spectrometry analysis, and matching against a transcriptome database obtained from the venom gland [22]. All contigs identified through this approach demonstrated high similarity with *Sus crofa* hyaluronidase-1 from a BLAST search in the Uniprot database (Version 46). A complete description of the proteomic results obtained from *C. consors* venom will be published elsewhere. The present results indicated that *C. consors* DV contained at least 10 Hyal isoforms with different pIs, of which five stained particularly intensely. Interestingly, from the nine protein spots on the IV 2-DE gel, eight were assigned to Hyals that shared similar molecular mass range and pI characteristics. Relative staining intensity between different Hyal isoforms was similar in DV and IV.

**Figure 2:**
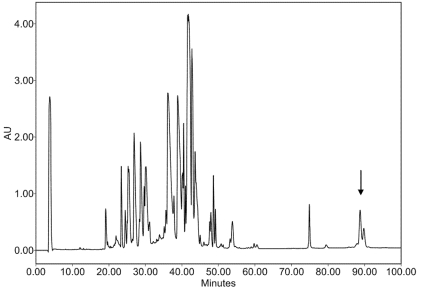
HPLC chromatogram with UV detection at λ = 214 nm of a standard pool of *C. consors* injectable venom (gradient of 1% solvent B (90% ACN/0.1% TFA in water) per minute, starting from 100% of solvent A (0.1% TFA in water)). Location of the peak assigned as Hyal is indicated by an arrow.

**Figure 3:**
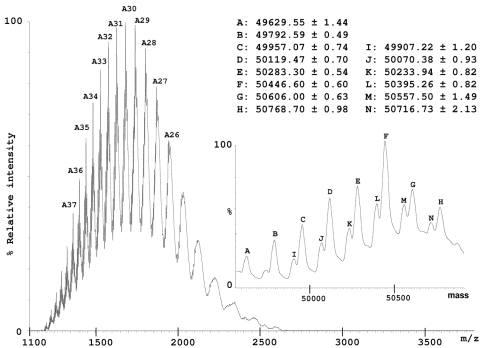
ESI-MS spectrum of all Hyal isoforms detected in the injectable venom with their average molecular masses. Inset shows the full mass transform spectrum. Two sets of masses could be identified, each showing mass differences of 162 Da, corresponding to Hex groups.

In addition to the 2-DE gel approach, a HPLC method was used to separate IV components. The IV sample resulting from a one-month period collection on numerous specimens provided a relatively stable protein profile over a period of nearly two years. This is in marked contrast with individual specimen venoms, where extreme variation in molecular composition can been observed [23]. A large peak eluting at the most hydrophobic part of the chromatogram (Figure 2) displayed numerous signals in electrospray ionization mass spectrometry (ESI-MS) analysis that could be assigned to large proteins in the 50 kDa molecular mass range. Detailed interpretation of the spectrum indicated at least two primary molecular entities differing by 50 Da (±1 Da), each with series of mass differences corresponding to *N*-acetyl-hexosamine (HexNAc, 203 Da) or hexose (Hex, 162 Da) moieties (Figure 3).

The DV protein profile of *C. consors* appeared complex and only a few proteins were selected for the injectable venom, including numerous Hyal isoforms. The expression of Conohyal-Cn1 is thus not only demonstrated in the venom apparatus, but its use as a venom component injected into the prey is also supported. The discovery of Hyals from a cone snail venom, and to a larger extent in the Mollusca phylum, partly confirms and completes the pioneering work performed by Sutherland and Lane [24] in *Octopus* venom. At the time, the authors described Hyal activity without being able to isolate and characterize the protein itself. Additionally, the discovery of cone snail venom Hyals is not fully unexpected as most other venoms contain this enzyme family. Indeed, complete or partial Hyal sequences have been determined from the venoms of snakes [25,26], anguimorpha lizards [27], scorpions [28], spiders (Richardson *et al.*, Uniprot accession number P86274), fish [29,30,31], and hymenopterans [32,33]. Thus, Hyals appear to have a role in a very large number of venoms, from vertebrates to invertebrates. 

**Figure 4:**
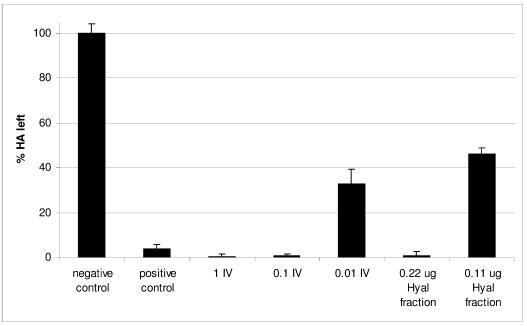
Hyaluronic acid (HA) degrading activity of injectable venom and purified Hyal fraction compared to negative control and bovine testicular hyaluronidase (5U) as a positive control. Activity is inversely proportional to the percentage of non-degraded HA, the turbidity of which was measured by absorbance at 400 nm. 1 IV is the quantity of crude venom equivalent to one sting, 0.1 IV is equivalent to 10% of a sting and 0.01 IV to 1%.

*C. consors* venom Hyal activity was first evaluated using the complete IV. To limit individual discrepancies, a pool of six crude freshly milked venoms was tested. Quantities equivalent to one injection and to 1/10 injection were found to be 100% active (Figure 4). Further dilution to 1/100 of the equivalent of one injection still produced hyaluronic acid (HA) degradation, but to a lesser extent (32.9% ± 6.45 of HA left). In a second step, the specific activity of the Hyal fraction purified by size exclusion chromatography was determined. Using bovine testicular hyaluronidase (BTH) as a standard (Supplementary Figure S1), the specific activity of the *C. consors* Hyal fraction was estimated to be 19,372 ± 1,224 U/mg (given the fact that 0.11 μg degraded 54% of HA, Figure 4).

*C. consors* Hyal fraction demonstrated a significant enzymatic activity in degrading hyaluronan, with a specific activity comparable to the Hyals found in scorpion venom [34,35]. By degrading the glycosaminoglycans that partly form extracellular matrices, venom Hyals are thought to improve and accelerate prey digestion. To some extent, degradation of the extracellular matrix by these enzymes also allows a rapid spread of other venom components during the envenomation process. For example, an increase in the efficiency of myotoxic phospholipase A_2_ and hemorrhagic metalloproteases has been reported in the presence of Hyals from snakes and spiders [36,37]. Venom diffusion and tissue degradation could well explain the presence of such enzymes in the venoms of a wide variety of animals. It is particularly notable that the spreading factor property of Hyals has already been applied to the medicinal area to improve the efficiency of other drugs [38,39,40], and new therapeutic strategies are emerging [41], with some applications currently in clinical trials [42].

### 2.2. Characterization of Conohyal-Cn1

**Figure 5:**
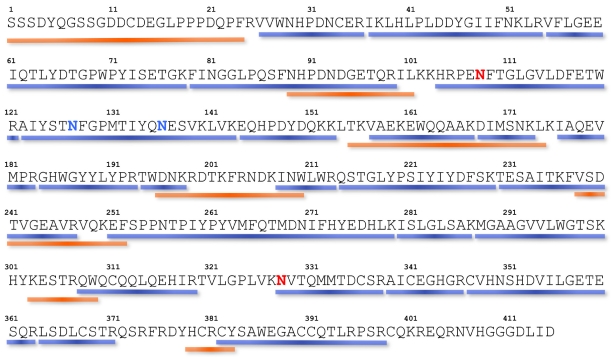
Sequence coverage and glycosylation sites of Conohyal-Cn1. Fragments were identified by reduction, alkylation and enzyme digestion of the UV-HPLC peak indicated in Figure 2. Sequence parts underlined in blue and orange correspond to tryptic and chymotryptic fragments, respectively. Unambiguous N-glycosylation sites are located at positions 108 and 328 (in bold red). Additional N-glycosylation was observed in fragment 122–153, with two possible sites (in bold blue). The first 10 amino acids on the N-terminus were also confirmed by Edman sequencing.

Material from the peak marked with an arrow (Figure 2) was submitted to reduction, alkylation and digestions by trypsin and chymotrypsin. The digestion mixtures were analyzed by ESI-MS and tandem mass spectrometry. Resulting MS/MS data were matched against the complete set of nucleotide sequences obtained from the transcriptome of *C. consors* venom glands using the Phenyx software (GeneBio, Geneva, Switzerland). A full-length nucleotide sequence was unambiguously matched with the trypsin and chymotrypsin digests, with scores of 154.26 and 50.62, respectively. This transcript (GenBank Accession Number: JN697596) was then selected and final manual interpretation was performed using unmatched MS/MS data. Using this approach, a few additional digestion fragments could be assigned, mainly because of previous errors inherent to the automatic determination of the parent ion signal values during the transformation process made on the raw MS/MS data using the MaxEnt3 module of the MassLynx software (Waters-Micromass, Manchester, UK). Altogether, a preliminary coverage of 70% of the expected complete sequence was obtained (Figure 5). The protein N-terminus could also successfully be determined by 10 cycles of Edman sequencing and strictly corresponded to the first 10 amino acids of the transcript. A similar transcript, named Conohyal-Ad1 (GenBank Accession Number: JN697597), was detected from a transcriptome of *C. adamsonii* venom gland, a species that is also a fish-hunting cone snail, from a different clade to *C. consors*.

Interestingly, proteomic data from a tryptic fragment deriving from Conohyal-Cn1 demonstrated a clear mismatch at position 45 of the expected sequence from the transcript (Figure 5). A glutamate (Glu) residue could be detected, whereas an aspartate (Asp) residue was coded in the nucleotide sequence. It must be noted that some other incomplete transcripts from the venom gland contained a Glu residue at this position, thus indicating the presence of a variant isoform at this amino acid position. An O-methylation of the aspartate residue cannot be completely ruled out as it could also explain the mass discrepancy observed but this PTM has never been described among *Conus* species so far. In addition, MS/MS data analysis demonstrated the presence of oxonium ions of Hex at *m/z* 163 and of HexNAc at *m/z* 204 in the spectra of several fragments that were further examined in more detail.

**Figure 6:**
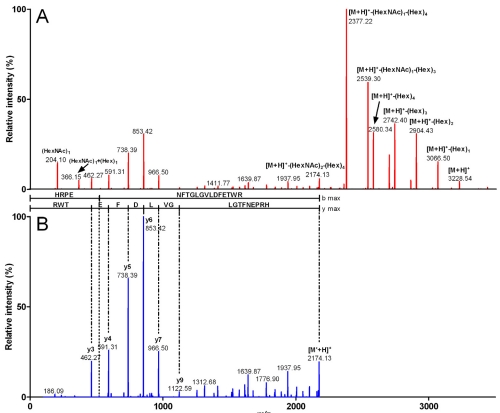
ESI-MS/MS spectra of a glycosylated fragment from Conohyal-Cn1. The ion at *m/z* 807.8 and corresponding to a 4x-charged species was fragmented by collision-activated dissociation. The singly-charged *m/z* MS/MS spectrum (A) revealed characteristic signals with mass differences of 162 Da (Hex) and/or 203 Da (HexNAc). Manual interpretation of the spectrum indicated the presence of (HexNAc)_2_(Hex)_4_, information that was used to generate the ESI-MS/MS spectrum in (B) where all Hex-related signals are removed. The final spectrum allowed a correct peptide assignment as the tryptic fragment 104–121.

Sequencing processes using mass spectrometry instruments software (for *de novo* sequencing) or peptide identification and sequencing software (such as Mascot from Matrix Sciences or Phenyx from GeneBio) can be complicated by post-translational modifications (PTMs), especially glycosylations. Indeed, such PTMs can occur on several different residues, and the different glycans can assemble to result in very complex structures and numerous molecular mass possibilities for the fragment. Furthermore, during the collision-induced dissociation process, these sugars preferentially fragment one by one or in clusters and contaminate the MS/MS mass spectrum with very high sugar signals. When using collision-activated dissociation, such spectra are usually considered useless in obtaining any amino acid sequence information [43,44]. A semi-automatic in-house program was thus created to help identify and sequence glycosylated peptides or proteins. After manual interpretation for determining the probable number and nature of sugars, the program recalculates the complete mass spectra files as well as the sugar-free monoisotopic mass of the sequence. These files can then be uploaded in MS data analysis software, or used in automatic peptide identification and sequencing software for rapid identification (Figure 6).

All MS/MS data showing characteristic Hex^+^ or HexNAc^+^ oxonium ion signals were treated as described above, and several fragments could be newly assigned with the number and type of sugars (Table 1). The glycosylation heterogeneity observed for each peptide is in agreement with the numerous Conohyal-Cn1 isoforms detected in IV. It must be noted that all glycoforms were detected at different retention times during the liquid chromatography analysis, thus excluding any in-source fragmentation of a single precursor sugar group. Two glycosylation sites at asparagine (Asn) position 108 and Asn328 could be identified, based on the fact that no O-glycosylated fragments were ever characterized in Hyals. However, glycosylation at serine and threonine positions 110, 111, 125, 126, 132 and 138 cannot be completely ruled out. A third glycosylation position could be attributed to Asn127 and/or Asn136, as this fragment contains two Asn residues. Theoretically, chymotryptic fragments (cleavage at Phe128 or at Tyr134) should have provided the precise location of the glycosylation site, but none of these fragments were detected. A fourth glycosylated fragment remained unidentified, possibly corresponding to a sequence deriving from a Conohyal-Cn1 isoform. Finally, the protein sequence coverage corresponded to 90% of the transcript sequence (Figure 5).

**Table 1:** List of tryptic fragments from Conohyal-Cn1 identified with a glycosylation pattern by ESI-MS/MS. Residue B accounts for carboxyamidomethyl-Cys, (m) stands for the protonated molecular mass and (z) corresponds to the ion charge state.

Fragment mass (Da)	*m/z* (z)	Peptide sequence	Sequence mass (Da)	Glycosylation	Glycosylation mass (Da)
2,557.84	853.62 (3)	NVTQMMTDBSR	1,341.45	(HexNAc)_2_ (Hex)_5_	1,216.39
2,233.74	1,117.87 (2)	NVTQMMTDBSR	1,341.45	(HexNAc)_2_ (Hex)_3_	892.29
2,071.70	1,036.85 (2)	NVTQMMTDBSR	1,341.46	(HexNAc)_2_ (Hex)_2_	730.26
3,540.30	1,181.10 (3)	AIYSTNFGPMTIYQNESVK	2,161.83	(HexNAc)_2_ (Hex)_6_	1,378.47
3,378.12	1,127.04 (3)	AIYSTNFGPMTIYQNESVK	2,161.73	(HexNAc)_2_ (Hex)_5_	1,216.39
3,054.09	1,019.03 (3)	AIYSTNFGPMTIYQNESVK	2,161.8	(HexNAc)_2_ (Hex)_3_	892.29
2,892.14	965.05 (3)	AIYSTNFGPMTIYQNESVK	2,161.88	(HexNAc)_2_ (Hex)_2_	730.26
3,916.49	980.12 (4)	HRPENFTGLGVLDFETWR	2,172.88	(HexNAc)_3_ (Hex)_7_	1,743.61
3,551.27	888.81 (4)	HRPENFTGLGVLDFETWR	2,172.8	(HexNAc)_2_ (Hex)_6_	1,378.47
3,227.22	807.81 (4)	HRPENFTGLGVLDFETWR	2,172.86	(HexNAc)_2_ (Hex)_4_	1,054.36

In spite of the relatively high number of Hyal sequences known to date (119 in the glycoside hydrolase family 56), only four sequences have been thoroughly investigated at the protein level, where a few positions have been shown to bear *N*-glycosylation groups (Supplementary Figure S2). The data obtained with Conohyal-Cn1 clearly demonstrated that this PTM is extensively present in the protein. Further, two glycosylation locations could be evidenced in the sequence, as well as a potential third one. Such a PTM has already been identified in several cone snail venom components, including contulakin-G [45], CcTx [46], and k-SIVA [47]. In the case of Conohyal-Cn1, data indicated the presence of Hex and HexNac moieties, but without allowing the exact sugar conformation. In addition to increasing the hydrophilic nature of the compounds, the glycosyl part usually remains necessary to keep the full activity of the molecule, thus implying a pivotal role of this group in functional interaction [45]. The role of glycosylation groups in Conohyal-Cn1 may also be important, for example as a first interaction with the substrate. Analysis of the 3D-model supports this hypothesis, with the glycosylated residues pointing to spatially very different directions to act as potential substrate sensors.

### 2.3. Structure Modeling

**Figure 7:**
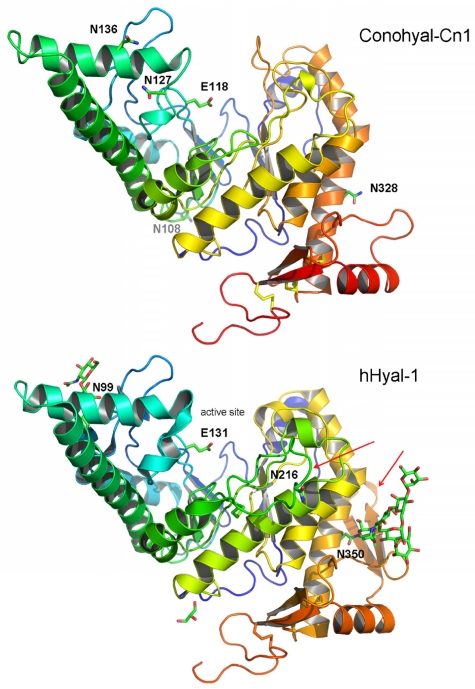
Homology model of Conohyal-Cn1 and X-ray structure of human Hyaluronidase-1 with display of the glycosylation and active sites. Glycosylated position Asn328 in Conohyal-Cn1 is equivalent to Asn350 in hHyal-1. Sugar location in Asn108 for Conohyal-Cn1 has no similarity with the human isoform. Other glycosylation sites include positions Asn127 and/or Asn136 in Conohyal-Cn1 and similarity is shared at position Asn99 for hHyal-1. Spatial position of active sites appears well conserved between the two Hyals. Major structural differences from the homology model of Conohyal-Cn1 and X-ray structure of hHyal-1 are the lack of two loops in Conohyal-Cn1 compared with hHyal-1 (indicated by red arrows, loops 1 and 2).

The recently solved structure of human Hyaluronidase-1 (hHyal-1) revealed a catalytic domain similar to that of bee venom Hyal (Phe22–Thr352) and a novel epidermal growth factor (EGF)-like domain (Ser353–Trp435) [48]. Both domains are conserved in Conohyal-Cn1, corresponding to sequence residues Asp14–Thr330 and Gln331–Ser397, respectively. Homology model of Conohyal-Cn1 shows that the catalytic domain contains equivalent residues for most of the hHyal-1 active site residues, including the key Glu131 (corresponding to Glu118 in Conohyal-Cn1, Figure 7). One significant structural difference resides in loop 1, which appears to be 11 residues shorter. Similarly, the EGF-like domain (loop 2) of Conohyal-Cn1 is truncated by 15 residues compared with hHyal-1. Yet, these modifications are not expected to lead to major deviations from the hHyal-1 structure, as they are located on loops that do not participate in/or interact with critical domains. Indeed, while loop 2 is clearly separate from the catalytic site, loop 1 stands on one side of the catalytic region, but ~14 Å away from Glu118 at the closest point. In addition, four disulfide bonds are conserved between Conohyal-Cn1 and hHyal-1, strongly suggesting a very similar overall fold for both proteins. Relative glycosylation positions demonstrate similar location between Asn136/Asn99 and Asn328/Asn350 (Conohyal-Cn1/hHyal-1), which would make Asn136 a very plausible glycosylation site *versus* Asn127. It can also be pointed out that potential glycosylation at position Asn136 would follow the N-X-S/T prediction guide for glycosylation whereas Asn127 would not [5,49]. Finally, glycosylation of Asn108 stands below the active site with no experimental equivalent on hHyal-1. Overall, it is interesting to note that glycosylation sites all point towards spatially complete different directions.

The sequence of Conohyal-Cn1 clearly shares elevated similarity with other Hyals of the hydrolase-type (EC 3.2.1.35), grouped into the glycoside hydrolase family 56 in the CAZy database [50]. In this family of vertebrate Hyal hydrolases, the catalytic Glu residue is located at position 131 (in reference to hHyal-1), corresponding to Glu118 in Conohyal-Cn1 (Supplementary Figure 2). Four important residues for the interaction with the substrate are also strictly conserved, *i.e.*, Asp116, Tyr189, Tyr223 and Trp296. Such residues are also widely conserved among the various vertebrate-like Hyals. These are thought to serve as positioning residues allowing the correct conformation of the *N*-acetyl-D-glucosamine moiety of hyaluronan for the subsequent catalytic reaction [21]. As can be observed from the 3D model based on hHyal-1, the positioning of important residues, as well as the glycosylation sites, are very well conserved in Conohyal-Cn1.

### 2.4. Phylogenetic Analysis

**Figure 8:**
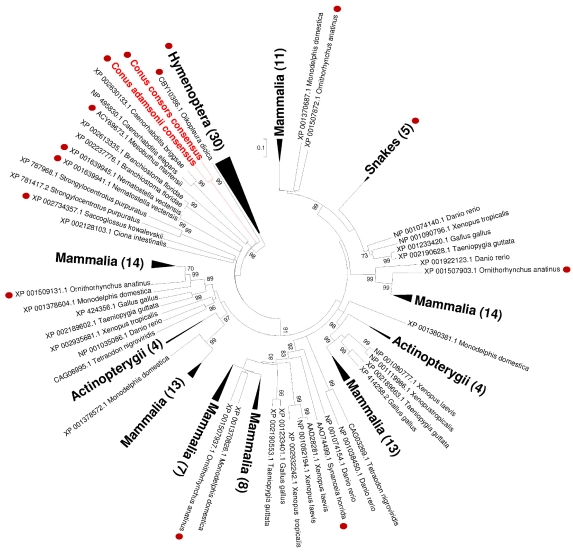
Evolutionary relationships of Hyal sequences using Maximum Likelihood method. Statistically supported clades, including at least five sequences from a monophyletic taxonomic group are collapsed in a same triangle to facilitate tree overview. The surface of each triangle is proportional to the number of sequences in the identified clade. The number of sequences is indicated under the caption of each group. Bootstrap values above 70% are indicated on each node. Outgroup composed by hymenopteran sequences is indicated by a dashed-grey triangle. Sequences are labeled using the Genbank accession number followed by the name of the species. The red dot indicates a putative venom function for a given sequence or in a given clade.

Phylogenetic analysis was performed on nearly 25% of the initial alignment, which include 177 protein sequences from a wide range of taxonomic groups. The best model calculated by ProtTest 3 according to likelihood ratio is LG [51], including invariant position parameter and gamma distribution variation of substitution rates among sites. Groups of hymenoptera Hyals were chosen to root the tree. According to results described in a previous publication [52], we identified many mammalian Hyal clades originating from ancient gene duplication events (Figure 8). This tree shows that independent convergent recruitment of Hyals with a putative venom function occurred in different taxonomic groups. Hyal sequences of the two cone snail species group together and are clearly separated from other clades. This position could come from long branch attraction phenomenon, which is common for highly divergent sequences.

With regard to other Hyal representatives, the two cone snail sequences reported here display a significant divergence, as can be noted from the phylogenetic construct (Figure 8). The unique Hyals of the Mollusca phylum appear well detached from all other clades, underlining that *Conus* venom Hyal genes probably evolved from a common ancestor, independently from other phyla. Thus, expression and use of this protein family in cone snail venoms add support in favor of an independent recruitment of Hyals in venomous animals. In addition, Conohyal-Cn1 and Conohyal-Ad1 sequences display 93% identity, in agreement with the close phylogenetic relationship between *C. consors* and *C. adamsonii*, belonging to the *Pionoconus* and *Textilia* clades, respectively [53]. It will certainly be interesting to investigate other groups of fish-hunting *Conus* species, such as the *Gastridium* and *Chelyconus* clades. Presence of Hyals in mollusc-hunting species is questionable, as a recent venom gland transcriptome of *C. marmoreus* failed to reveal any Hyals (personal observation from S. D.). In addition, a recent proteomic survey of the whole venom gland of the mollusc-hunting species *C. victoriae* did not mention any Hyal-like compounds [54]. Current data thus suggest that Hyals could be restricted to specific *Conus* clades, preferentially those with a fish diet, although further studies would be needed to confirm this hypothesis.

## 3. Experimental Section

### 3.1. Chemicals

Acetonitrile (ACN, from Fisher Scientific Ltd., Loughborough, UK) and formic acid (Acros Organics, Geel, Belgium) were of HPLC grade or higher. Purified deionized water was obtained using a Milli-Q system (Millipore Corp., Billerica, MA, USA). When required, each solvent was filtered and sonicated before use. Imobiline Drystrips (pH 3–11 NL, 7 and 24 cm), carrier ampholytes and DeStreak Reagent were from GE Healthcare (Uppsala, Sweden). LC-MS reagents 0.1% formic acid and 0.1% formic acid in acetonitrile were from J.T.Baker (Deventer, The Netherlands). Bovine testicular hyaluronidase, HA and hexadecyltrimethylammonium bromide were purchased from Sigma-Aldrich (St Louis, MO, USA).

### 3.2. Sample Preparations

All specimens of *C. consors* used for this study were collected from one colony in the Chesterfield Islands (New Caledonia) during the CONFIELD-I scientific expedition in June 2007. A single specimen of *C. adamsonii* was found during the CONPOL-I campaign at Nuku-Hiva (Marquesas archipelago) in November 2007. Dissected venom was a pool of crude venom obtained from several individual specimens dissected on ice, and from which the venom duct was removed. The venom duct content was diluted in 500 μL of 10% acetic acid, freeze-dried and stored at -80 °C until use. Venom duct samples were stored in RNALater according to the manufacturer’s protocol (Qiagen). Total RNA was extracted with TRIzol® reagent using standard protocols (Invitrogen).

### 3.3. Injectable Venom Fractionation

Injectable venom was obtained following a milking procedure adapted from Hopkins and colleagues [55]. A standard pool of IV consisted of approximately 70 milkings from 35–40 specimens over one month. The sample was fractionated by RP-HPLC using an Alliance HT 2795 (Waters, Milford, MA, USA) separation module fitted with a Waters 996 Photodiode Array Detector and operated with the Waters Millenium 4.0 software. RP-HPLC was performed using a 218TP510 C18 RP column (10x250 mm, 5μm, Vydac, Hesperia, CA, USA). The flow rate was set to 0.8 mL/min with a gradient of 1% solvent B (90% ACN/0.1% TFA in water) per minute, starting from 100% of solvent A (0.1% TFA in water). Collected fractions were lyophilized and stored at −20 °C until use.

### 3.4. cDNA Library Construction, 454 Sequencing and Assembly

First, 5 μg of total RNA was used to construct a cDNA library. RNA quality was assessed in a Bioanalyzer 2100 (Agilent-Bonsai Technologies). 5 μg of full-length double-stranded cDNA was then processed by the standard Genome Sequencer library-preparation method using the 454 DNA Library Preparation Kit (Titanium chemistry) to generate single-stranded DNA ready for emulsion PCR (emPCR™). The cDNA library was then nebulized according to the fragmentation process used in the standard Genome Sequencer shotgun library preparation procedure. The cDNA library was sequenced according to GS FLX technology (454/Roche). The short-reads from the sequencing were assembled by MIRA version 2.9.25 using enhanced 454 parameters.

### 3.5. Two-Dimensional Gel Electrophoresis (2-DE)

Lyophilized DV was dissolved in milliQ water, and the water-soluble fraction was lyophilized and used for 2-DE experiments. IV was completely soluble in the miliQ water. For the first dimension, 7 cm immobilized pH gradient (IPG) strips 3–11 NL (GE Healthcare) were passively rehydrated overnight with a sample (10 μg of DV or 20 μg of IV, venom weight based on an aliquote of a dried 1 mg batch quantified by a Mettler Toledo scales.) in 125 μL of rehydration buffer (7 M urea, 2 M thiourea, 30 mM Tris, 0.25% (w/v) ASB-14, 3.0% (w/v) CHAPS, 0.002% (w/v) Bromphenol Blue, 0.75% ampholytes and 12 μL/mL DeStreak Reagent). IEF was performed on Ettan IPGphor II (GE Healthcare) at 20°C using the following sequential steps: 300 V for 45 min; 300–1000 V gradient for 30 min; 1000–5000 V gradient for 1.20 h; 5000 V to a total of 6000 Vh. The current was restricted to 50 μA/strip. For the second dimension, SDS-PAGE, the focused IPG strips were reduced with 65 mM DTT in 75 mM Tris-HCl buffer containing 6 M urea, 4% (w/v) SDS, 30% (v/v) glycerol, 0.002% (w/v) Bromphenol Blue, and then alkylated with 135 mM iodoacetamide in the same buffer. The equilibrated strips were applied on 10% (w/v) polyacrylamide gels cast in a gel buffer containing 150 g Tris in 0.6 M HCl and SDS PAGE ran in Tris/Taurine buffer system at 10 mA/gel as described [56]. Protein spots were detected by silver staining. For preparative analysis of DV, a 24 cm IPG strip 3–11 NL was passively rehydrated overnight with 350 μg sample in 450 μL of rehydration buffer and IEF ran using the following conditions: 150 V for 3 h, 300 V for 3 h, 300–1000 V gradient for 6 h, 1000–10,000 V gradient for 1 h and 10,000 V for 3 h, a total of 40,000 Vh and with maximal current of 75 μA/strip. The focused proteins were reduced, alkylated and analyzed in the second dimension SDS-PAGE as described above. This was carried out using the Ettan DALT six unit (GE Healthcare) at 15 °C, applying 2 W/gel for 1 h and 17 W/gel for a further 5 h. Protein spots were reverse-stained with imidazole-zinc [57]. Images of gels were acquired using Image Scanner and LabScan 5 software (GE Healthcare, Amersham Biosciences), and analyzed with Image Master 2D Platinum 6.0 software (GE Healthcare, Amersham Biosciences). The spots were picked using Ettan spotpicker (GE Healthcare, Amersham Biosciences) and unstained in 70% (v/v) Tris/Gly (50 mM/0.3 M) and 30% (v/v) acetonitrile. The gel pieces were then consecutively washed three times with 10 mM NH_4_HCO_3_ and 10 mM NH_4_HCO_3_/ACN (1:1, v/v), followed by 100% acetonitrile and dried under vacuum. Proteins in gels were digested with trypsin (Trypsin, Proteomics Grade, Sigma-Aldrich) in 25 mM NH_4_HCO_3_ at 37 °C overnight. Peptides were extracted with 50% (v/v) ACN/5% (v/v) formic acid, concentrated in vacuum to 15 μL and stored at −20 °C.

### 3.6. Mass Spectrometry (MS and MS/MS) on 2-DE Spots

LC-MS/MS analyses were performed on an ion trap mass spectrometer 1200 series HPLC-Chip-LC/MSD Trap XCT Ultra (Agilent Technologies, Waldbronn, Germany) with an ESI source operating in positive mode, controlled by ChemStation LC 3D systems Rev. B.01.03 SR1 (Agilent Technologies, Santa Clara, CA, USA) and LC/MSD Trap Control software version 6.0 (Bruker Daltonik GmbH, Bremen, Germany). An HPLC-Chip (Agilent Technologies, Waldbronn, Germany) is comprised of a 40 nL enrichment column and a 43 mm x 75 µm analytical column packed with Zorbax 300SB-C18 5 µm particles. Peptides were loaded onto the enrichment column with 97% solvent A (0.1% formic acid in water) and 3% B (0.1% formic acid in ACN) at 4 µl/min. They were eluted from the analytical column with a gradient from 3% B to 50% B in 41 min, followed by a steep gradient to 90% B in 1 min at a flow rate of 0.35 µl/min. MS acquisitions were carried out from 400 to 2,200 m/z followed by MS/MS scans of the five most abundant ions in each MS scan. Spectrum Mill MS Proteomics Workbench Rev A.03.03.084 SR4 software (Agilent Technologies, Santa Clara, CA, USA) was used to search MS and MS/MS spectra against the *C. consors* transcriptome data file. The database was searched for tryptic peptides with a mass tolerance of ±2.5 Da for the precursor ions and a tolerance of ±0.7 Da for the fragment ions. Two missed cleavages were allowed. Autovalidation in the peptide mode was performed using a score threshold of 9 and scored peak intensity of 80% for +2 and +3 precursor ion charges. Forward minus reversed score threshold and rank 1 minus rank 2 score threshold were set to 2. All protein hits found in a distinct database search by Spectrum Mill are non-redundant.

### 3.7. Protein Biochemistry and Edman Sequencing

Lyophilized IV fractions of interest were suspended in 70% ACN at a concentration of 1 mg/mL. Disulfide bridges were reduced by incubation at 55 °C for 2 h in presence of ammonium bicarbonate and DTT 4.5 mM. Free SH groups were then alkylated by addition of 10 μL iodoacetamide 100 mM (15 min, RT). Excess of iodoacetamide was neutralized with 10 μL of cysteine 200 mM. Two different digestions were then carried out, using trypsin and chymotrypsin. In both cases, 50 μL of bicarbonate 2% were added and enzyme concentration was adjusted to have an enzyme/protein ratio of 1:20. Mixtures where then incubated at 37 °C for 6 h before quenching with 5 μL formic acid 20%.

Edman sequencing was performed using a Procise 491cLC sequencer (Applied Biosystem, USA). Approximately 1 nmol of the fraction of interest was deposited on a Biobrene-fixed filter and 15 sequencing cycles were carried out for N-terminal residue identification.

### 3.8. Mass Spectrometry on HPLC Fractions

ESI-MS analyses were performed on a Q-TOF Ultima mass spectrometer (Waters-Micromass, Manchester, UK) fitted with its standard ESI source and operated in positive mode under the control of MassLynx 4.0 software (Waters-Micromass). For Hyal native fraction analysis, sample loop injection was performed, and acquisitions were carried out from 100 to 4,000 *m/z* with infusion of H_2_O/MeOH/FA 50:50:0.2 (v/v/v) at a flow rate of 20 μL/min. The instrument was externally calibrated using NaCsI. Digestion fragments were analyzed using the same instrument as above by LC-ESI-MS, with data directed acquisition of MS/MS. RP-HPLC was performed using an Alliance HT 2795 (Waters, Milford, MA, USA) separation module fitted with a Waters 996 Photodiode Array Detector and operated with the Waters Millenium 4.0 software. A Waters Atlantis T3 column (1 × 150 mm, 3 μm) was used for separation. The flow rate was set to 0.05 mL/min with a gradient of 1% solvent B (90% ACN/0.1% FA in water) per minute, starting from 100% of solvent A (0.1% FA in water). The instrument was externally calibrated using Glu-fibrinopeptide. MS acquisitions were carried out from 100 to 1,800 *m/z*. MS/MS survey was set up to adjust collision energy depending on the charge state of the fragmented ion using automated charge state recognition (from 1 to 4 charge state). All MS data were manually interpreted using the Biolynx option of MassLynx 4.0 software (Waters-Micromass). MS/MS data were deconvoluted into a singly-charged *m/z* axis using the MaxEnt3 (Waters-Micromass). Files were then submitted to Phenyx v.2.6.2 (Genebio, Geneva, Switzerland) and matched against the transcriptome datafile. MS/MS spectrum files that showed glycosylation signals and did not match directly to the transcriptome datafile were submitted to an in-house progam developed in PERL language. After a manual identification of the nature and number of sugars, the program automatically suppresses all the sugar signals from the spectrum file with a user-defined ppm margin. The transformed MS/MS spectrum files were then resubmitted to Phenyx for matching. Results were manually assessed and checked using Biolynx and the consensus transcript sequence for MS and MS/MS match. Sequence attribution was also carried out with the help of MassSeq (Waters-Micromass).

### 3.9. Enzymatic Activity

In order to avoid acidic conditions, which may deactivate the enzyme, Conohyal-Cn1 and its isoforms were purified from 50 μL of *C. consors* IV by size exclusion chromatography using an Alliance HT 2795 (Waters, Milford, MA, USA) separation module fitted with a Waters 487 dual wavelength detector and operated with the Waters Millenium 4.0 software. The column used was a BioSep-SEC-S 2000 (300x7.8 mm, Phenomenex, Torrance, CA, USA). The flow rate was set to 0.5 mL/min with an isocratic composition of acetate buffer (0.05 M sodium acetate, 0.15 M NaCl, pH adjusted to 5 with acetic acid). The fraction containing the Hyals was checked by LC-ESI-MS for the presence of proteins in the 50 kDa mass range. Enzymatic activity was determined turbidometrically [58]. Briefly, mixtures (final volume 0.5 mL) containing 50 μg of HA with test solutions in acetate buffer (0.05 M sodium acetate, 0.15 M NaCl, pH adjusted to 5 with acetic acid) were incubated 15 min at 37 °C. The reaction was quenched by the addition of 1 mL of 2.5% hexadecyltrimethylammonium bromide in 2% NaOH and absorbance was monitored at 400 nm on a Spectramax M5 spectrophotometer (Molecular devices, Sunnyvale, CA, USA). A solution made of 0.5 mL of buffer with 1 mL of quenching solution was used as blank. Negative control (50 μg HA in buffer without enzyme) and positive control (BTH) were also included. Turbidity reducing activity was expressed as the percentage of remaining HA.

### 3.10. Homology Modeling

A three-dimensional model of Conohyal-Cn1 was built using the project mode of the SWISSMODEL online server [59]. Template identification revealed that the structure of hHyal-1 had the highest percentage of sequence identity (32%). Briefly, the FASTA sequence of Conohyal-Cn1 was loaded into the DEEPVIEW program. The structure of hHyal-1 (PDB code 2PE4) was then loaded, and its sequence automatically aligned with that of Conohyal-Cn1. Manual adjustments were necessary to increase the quality of alignment. Importantly, key residues, such as those in the catalytic domain as well as four disulfide bonds were conserved between the 2 sequences, strongly suggesting that both proteins have a similar architecture. The resulting model was minimized using the implemented GROMOS force fields and visualized in PYMOL.

### 3.11. Phylogenetic Analysis

The set of Hyal sequences was retrieved by keyword on the nr GenBank database and included the two Hyal contigs from *C. consors* and *C. adamsonii*. Alignments of matching sequences were performed with Muscle software [60] and manually adjusted if needed. Prior to phylogeny analysis, divergent and ambiguously aligned blocks were removed using Gblocks software [61]. The ProtTest 3 software was used to choose the best-fitted model for phylogeny analysis [62]. Phylogeny analyses were carried out using maximum likelihood method with the PhyML software [63]. and trees were visualized and annotated using the tree viewer of MEGA 5 [64]. Bootstrap support values were based on 100 replicates.

## 4. Conclusions

In contrast with the current opinion, our study evidenced the presence of high molecular mass proteins in a cone snail venom (injected fluid), thus expanding the molecular biodiversity of these complex mixtures. Combination of venom gland transcriptomics and venom proteomics efficiently afforded a complete primary structure assignment, including post-translational glycosylations, of the first hyaluronidase sequence in the Mollusca phylum. In addition to demonstrating the recruitment of this enzyme family in marine snail venom, its potent biological activity in degrading hyaluronic acid is reflecting an important role in the envenomation process. In allowing faster venom diffusion in the prey tissue, these enzymes magnify the potency of all venom conopeptides, allowing more efficient prey capture and digestion or defensive actions against predators. This eventually leads to the cone snail’s advantage in the evolutionary race with its prey. Overall, this work highlights a neglected protein family [65], not yet described in cone snail venoms. Furthermore, the molecular and physiological mechanisms underlying hyaluronidase recruitment in the injectable venom and their precise biological impact will likely deserve more investigations that will add valuable insights into the venomous function of these marine animals.

## Supplementary Files

Supplementary File 1: PDF-Document (PDF, 155 KB)
