# Supplementary material for: Recruitment of Glycosyl Hydrolase Proteins in a Cone Snail Venomous Arsenal: Further Insights into Biomolecular Features of Conus Venoms
Source: Mar Drugs. 2012 Jan 31;10(2):258–80. doi: 10.3390/md10020258 (PMC3296996; doi:10.3390/md10020258)
Supplement: Supplementary File 1: — PDF-Document (PDF, 155 KB) [file marinedrugs-10-00258-s001.pdf]

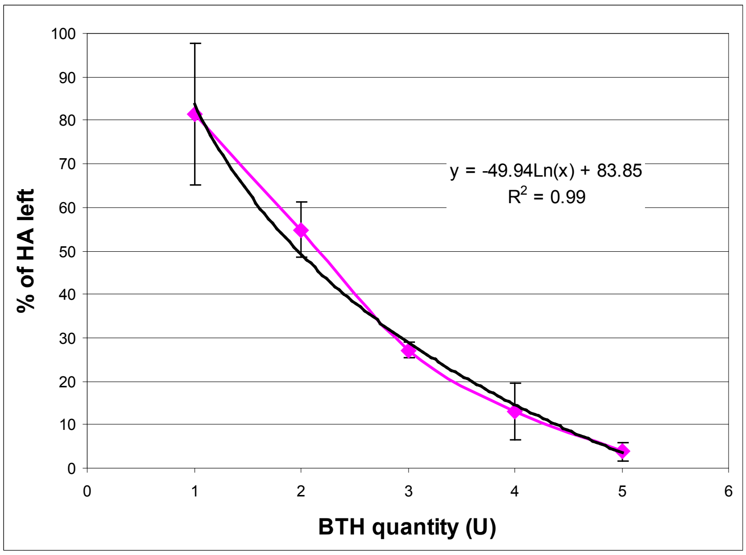

Supplementary Figure S1: percentage of hyaluronic acid (HA) left as a function of bovine testicular hyaluronidase quantity in U (calculated from the specific activity value provided by the supplier) and its trend curve using a logarithmic fit.

```
sp|Q12794|HYAL1_HUMAN    -------MAAHLLPICALFLTLLDMAQGFRG----PLLPNRPFTTVWNANTQWCLER-HG  48
sp|O35632|HYAL2_MOUSE    -------MRAGLGPIITLALVLEVAWAGELKPTAPPIFTGRPFVVAWNVPTQECAPR-HK  52
sp|Q08169|HUGA_APIME     MSRPLVITEGMMIGVLLMLAPINALLLGFVQSTPDNNKTVREFNVYWNVPTFMCHKYGLR  60
sp|P49370|HUGAA_VESVU    -----------------------------------SERPKRVFNIYWNVPTFMCHQYDLY  25
Conohyal-Cn1             -------------------SSSDYQGSSGDDCDEGLPPPDQPFRVVWNHPDNCERIK---  38
                                                               . : *   **

sp|Q12794|HYAL1_HUMAN    VDVDVSVFDVVANPGQTFRGPDMTIFYSSQLGTYPYYTPTG--EPVFGGLPQNASLIAHL  106
sp|O35632|HYAL2_MOUSE    VPLDLRAFDVKATPNEGFFNQNITTFYYDRLGLYPRFDAAG--TSVHGGVPQNGSLCAHL  110
sp|Q08169|HUGA_APIME     FEEVSEKYGILQNWMDKFRGEEIAILYDPGMFPALLKDPNGNVVARNGGVPQLGNLTKHL  120
sp|P49370|HUGAA_VESVU    FDEVTN-FNIKRNSKDDFQGDKIAIFYDPGEFPALLSLKDGKYKKRNGGVPQEGNITIHL  84
Conohyal-Cn1             LHLPLDDYGIIFNKLRVFLGEEIQTLYDTG--PWPYISETG--KFINGGLPQSFNHPDND  94
                         .      :.:  .    * .:  :*             *      **:**  .   :

sp|Q12794|HYAL1_HUMAN    ARTFQDILAAIPAPDFSGLAVIDWEAWRPRWAFNWDTKDIYRQRSRALVQAQHPDWPAPQ  166
sp|O35632|HYAL2_MOUSE    PMLKESVERYIQTQEPGGLAVIDWEEWRPVWVRNWQEKDVYRQSSRQLVASRHPDWPSDR  170
sp|Q08169|HUGA_APIME     QVFRDHLINQIPDKSFPGVGVIDFESWRPIFRQNWASLQPYKKLSVEVVRREHPFWDDQR  180
sp|P49370|HUGAA_VESVU    QKFIENLDKIYPNRNFSGIGVIDFERWRPIFRQNWGNMKIHKNFSIDLVRNEHPTWNKKM  144
Conohyal-Cn1             GETQRILKKHRPE-NFTGLGVLDFETWRAIYSTNFGPMTIYQNESVKLVKEQHPDYDQKK  153
                               :       .  *:.*:*:* **. :  *:     ::: *  :*  .** :

sp|Q12794|HYAL1_HUMAN    VEAVAQDQFQGAARAWMAGTLQLGRALRPRGLWGFYGFPDCYNYDFLS--PNYTGQCPSG  224
sp|O35632|HYAL2_MOUSE    VMKQAQYEFEFAARQFMLNTLRYVKAVRPQHLWGFYLFPDCYNHDYVQNWESYTGRCPDV  230
sp|Q08169|HUGA_APIME     VEQEAKRRFEKYGQLFMEETLKAAKRMRPAANWGYYAYPYCYNLTPNQ----PSAQCEAT  236
sp|P49370|HUGAA_VESVU    IELEASKRFEKYARFFMEETLKLAKKTRKQADWGYYGYPYCFNMSPNN----LVPECDVT  200
Conohyal-Cn1             LTKVAEKEWQQAAKDIMSNKLKIAQEVMPRGHWGYYLYPRTWDN-------------KRD  200
                         :   *. .::  .:  *  .*:  :       **:* :*  ::

sp|Q12794|HYAL1_HUMAN    IRAQNDQLGWLWGQSRALYPSIYMPAVLEGTGKSQMYVQHRVAEAFRVAVAAGD-PNLPV  283
sp|O35632|HYAL2_MOUSE    EVARNDQLAWLWAESTALFPSVYLDETLASSVHSRNFVSFRVREALRVAHTHHANHALPV  290
sp|Q08169|HUGA_APIME     TMQENDKMSWLFESEDVLLPSVYLRWNLTSG-ERVGLVGGRVKEALRIARQMTTSRKKVL  295
sp|P49370|HUGAA_VESVU    AMHENDKMSWLFNNQNVLLPSVYVRQELTPD-QRIGLVQGRVKEAVRISNNLKHS-PKVL  258
Conohyal-Cn1             TKFRNDKINWLWRQSTGLYPSIYIYDFSKTESAITKFVSDTVGEAVRVQKEFSP--PNTP  258
                            .**:: **: ..  * **:*:             *   * **.*:

sp|Q12794|HYAL1_HUMAN    LPYVQIFYDTTNHFLPLDELEHSLGESAAQGAAGVVLWVS---WENTRTKESCQAIKEYM  340
sp|O35632|HYAL2_MOUSE    YVFTRPTYTRGLTGLSQVDLISTIGESAALGSAGVIFWGD---SEDASSMETCQYLKNYL  347
sp|Q08169|HUGA_APIME     PYYWYKYQDRRDTDLSRADLEATLRKITDLGADGFIIWGS---SDDINTKAKCLQFREYL  352
sp|P49370|HUGAA_VESVU    SYWWYVYQDETNTFLTETDVKKTFQEIVINGGDGIIIWGS---SSDVNSLSKCKRLQDYL  315
Conohyal-Cn1             IYPYVMFQTMDNIFHYEDHLKISLGLSAKMGAAGVVLWGTSKHYKESTRQWQCQQLQEHI  318
                                           .:  ::   .  *. *.::*      .:      *  :::::

sp|Q12794|HYAL1_HUMAN    DTTLGPFILNVTSGALLCSQALCSGHGRCVRRTSHPKALLLLNPASFSIQ--LTPGGGPL  398
sp|O35632|HYAL2_MOUSE    TQLLVPYIVNVSWATQYCSWTQCHGHGRCVRRNPSANTFLHLNASSFRLVPGHTPSEPQL  407
sp|Q08169|HUGA_APIME     NNELGPAVKRIALNNNANDRLTVDVSVDQV------------------------------  382
sp|P49370|HUGAA_VESVU    LTVLGP----IAIN--------VTEAVN--------------------------------  331
Conohyal-Cn1             RTVLGPLVKNVTQMMTDCSRAICEGHGRCVHN---SHDVILGETESQRLSDLCSTRQSRF  375
                            * *    ::

sp|Q12794|HYAL1_HUMAN    SLRGALSLEDQAQMAVEFKCRCYPGWQAPWCER---------KSMW--------------  435
sp|O35632|HYAL2_MOUSE    RPEGQLSEADLNYLQKHFRCQCYLGWGGEQCQRNYKGAAGNASRAWAGSHLTSLLGLVAV  467
sp|Q08169|HUGA_APIME     ------------------------------------------------------------  382
sp|P49370|HUGAA_VESVU    ------------------------------------------------------------  331
Conohyal-Cn1             RDYHCRCYSAWEGACCQTLRPSRC---QKREQRNVHGGGDLID-----------------  415

sp|Q12794|HYAL1_HUMAN    ------  435
sp|O35632|HYAL2_MOUSE    ALTWTL  473
sp|Q08169|HUGA_APIME     ------  382
sp|P49370|HUGAA_VESVU    ------  331
Conohyal-Cn1             ------  415
```

Supplementary Figure S2: Comparison of amino acid sequences of hyaluronidases from human (Uniprot Accession code Q12794), mouse (Uniprot Accession code O35632), *Apis mellifera* (Uniprot Accession code Q08169), and *Vespula vulgaris* with the Conohyal-Cn1. For all sequences, the catalytic residue is highlighted in green, the positioning residues in blue, and the characterized glycosylation sites in orange. The Asn residue highlighted in pink in Conohyal-Cn1 sequence corresponds to a probable glycosylation site deduced from proteomic experiments and similarity with the established glycosylation site in the hyaluronidase from *Vespula vulgaris* venom.
